# Current Transport Mechanism in Palladium Schottky Contact on Si-Based Freestanding GaN

**DOI:** 10.3390/nano10020297

**Published:** 2020-02-10

**Authors:** Moonsang Lee, Chang Wan Ahn, Thi Kim Oanh Vu, Hyun Uk Lee, Yesul Jeong, Myung Gwan Hahm, Eun Kyu Kim, Sungsoo Park

**Affiliations:** 1 Research Center for Materials Analysis, Korea Basic Science Institute, Gwahak-ro 169-148, Yuseong-gu, Daejeon 34133, Korea; lms1015@kbsi.re.kr (M.L.); leeho@kbsi.re.kr (H.U.L.); 2 Department of Physics and Research Institute for Convergence of Basic Sciences, Hanyang University, Seoul 04763, Korea; anchw93@hanyang.ac.kr (C.W.A.); vuthikimoanh92@gmail.com (T.K.O.V.); 3 Busan Center, Korea Basic Science Institute, Busan 46742, Korea; ysjeong@kbsi.re.kr; 4 Department of Materials Science and Engineering, Inha University, 100 Inharo, Michuhol-gu, Incheon 22212, Korea; 5 Department of Science Education, Jeonju University, 303 Cheonjam-ro, Wansan-gu, Jeollabuk-do 303, Korea; 6 Analytical Laboratory of Advanced Ferroelectric Crystals, Jeonju University, 303 Cheonjam-ro, Wansan-gu, Jeollabuk-do 303, Korea

**Keywords:** freestanding GaN, HVPE, Schottky diodes, silicon, transport mechanism

## Abstract

In this study, the charge transport mechanism of Pd/Si-based FS-GaN Schottky diodes was investigated. A temperature-dependent current–voltage analysis revealed that the I-V characteristics of the diodes show a good rectifying behavior with a large ratio of 10^3^–10^5^ at the forward to reverse current at ±1 V. The interface states and non-interacting point defect complex between the Pd metal and FS-GaN crystals induced the inhomogeneity of the barrier height and large ideality factors. Furthermore, we revealed that the electronic conduction of the devices prefers the thermionic field emission (TFE) transport, not the thermionic emission (TE) model, over the entire measurement conditions. The investigation on deep level transient spectroscopy (DLTS) suggests that non-interacting point-defect-driven tunneling influences the charge transport. This investigation about charge transport paves the way to achieving next-generation optoelectronic applications using Si-based FS-GaN Schottky diodes.

## 1. Introduction

Gallium nitride (GaN)-related alloys hold great promise for futuristic optoelectronic applications, owing to their prominent physical properties [1,2,3,4]. To enhance the characteristics of GaN-based devices, the introduction of freestanding (FS) GaN substrates into the applicable devices is essential. To achieve high crystalline freestanding GaN crystals, a number of researchers have studied various growth methods [5,6,7,8,9,10]. Of these methods, the outstanding characteristics of hydride vapor phase epitaxy (HVPE) growth, with a high growth rate and a high crystalline growth capability, can provide significant advances to achieve freestanding GaN crystals with a large scalability and economic advantages [11]. However, challenges such as size limits (<6 inch diameter) and manufacturing expenditure have restricted the successful introduction of HVPE freestanding GaN wafers into the commercial community [12]. Recently, we realized Si-based homoepitaxial InGaN/GaN multi-quantum well (MQW) light-emitting diodes (LEDs) with a large scalability and a desirable production cost [13,14,15,16].

To optimize the optoelectronic device using Si-based FS-GaN, it is necessary to understand the electronic transport characteristics of Schottky contact on the Si-based freestanding GaN. Their exploration, however, has never been inspected. Even though the literatures explained the conduction mechanism of metal/freestanding GaN, it does not provide information on S-based FS Schottky barrier diodes.

In this paper, we explored the charge transport characteristics of the Pd/Schottky diode on Si-based freestanding GaN crystals, thus elucidating how these influence GaN-based device performance.

## 2. Materials and Methods

**Figure 1:**
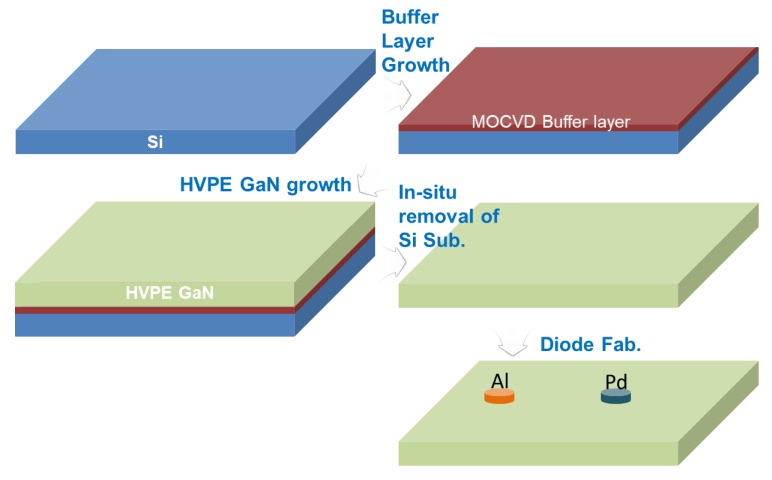
The fabrication procedure of the Pd/Si-based FS-GaN Schottky diode used in this study.

Si-based FS-GaN crystals were prepared by the in situ removal method of a Si substrate by HVPE. The details are described in [13]. For the electrical measurement of the Schottky diodes on Si-based FS-GaN, the ohmic and Schottky contacts were achieved. A 150 nm thick and 3 mm diameter Al ohmic contact was constructed on the Ga-face of the Si-based freestanding GaN, using a thermal evaporator (Infinity vacuum, Seoul, Korea). Subsequently, the Schottky contacts with 1.2 mm in diameter were formed by an electron beam evaporator (Sorona, Seoul, Korea) using Pd (80 nm) metal on the Ga-surface of the template, after which rapid thermal annealing in Ar ambient at 550 °C was employed. Al and Pd were deposited by a thermal evaporator and an e-beam evaporator under a vacuum level of 7 × 10^−6^ Torr using a metal mask with a hole, respectively. The deposition rate of both metals is 5 Å/sec. Figure 1 shows the detailed fabrication procedure. It was confirmed from the shape of the I-V curve that Al contact is ohmic. The electronic transport behaviors of the Schottky diodes were analyzed via temperature-dependent current–voltage (IV) characteristics in a temperature range from 220 K to 380 K. The dislocation density of Si-based FS-GaN was estimated to be 1 × 10^6^/cm^2^, evaluated by photoluminescence (PL) mapping analysis (not shown). Furthermore, a homemade DLTS system was used. The pulse voltage in the DLTS measurement was −2 V. The filling pulse widths used were 10 ms, 100 ms, and 500 ms, respectively. The DLTS data were obtained in the temperature range from 100 K to 420 K with increments of 0.1 K. Various emission rates with 3.66–0.93 Hz were used to find the DLTS signal. The interval between the filling pulse widths was 50 ms under all conditions.

## 3. Results and Discussion

**Figure 2:**
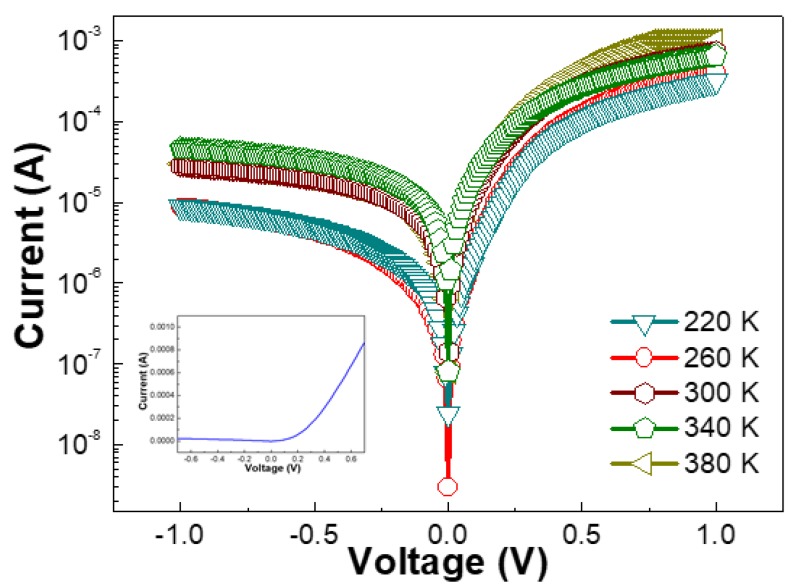
The *T-I–V* characteristics of the Pd Schottky diodes based on the freestanding GaN crystals peeled off from a Si substrate.

The temperature-dependent current–voltage (T-I-V) behaviors of the Pd/Si-based FS-GaN Schottky diodes are illustrated in Figure 2. One can clearly observe that the forward voltage in the T-I-V plots starts linearly at the initial voltage range (see the I-V curve at 300 K in the inset of Figure 2). However, the series resistance (R_s_) drives the distortion of the initial linearity over 0.3 V in the entire temperature range. Furthermore, it is noticeable that the charge flux increased by increasing the temperature, which is attributed to thermally generated current carriers [17]. On the other hand, the rectifying characteristics in the reverse bias clearly suggest the formation of Schottky diodes of the Pd/Si-based FS-GaN crystals in the whole temperature range. The large ratio of the forward to reverse current at ±1 V, spread over the range of 10^0^–10^2^, clearly proves the good rectification behavior of the diodes.

To shed light on the electronic transport characteristics of the diodes, the thermionic emission (TE) theory was applied to extract the electrical parameters of the devices as follows [18]:

(1) $$I=AA^{**}T^{2}exp\left(-\frac{q\Phi_{B}}{kT}\right)\left[exp\left(\frac{qV}{nkT}\right)-1\right)for\;V\;\geq \;3kT/q$$

(2) $$I_{0}=AA^{**}T^{2}\exp\left(-\frac{q\Phi_{B}}{kT}\right)$$

where *I*_0_ is the saturation current, *A* the contact area, A** the Richardson constant (26.4 A·cm^−2^·K^−2^ for n-type GaN), k the Boltzmann constant, *T* the absolute temperature, *q* the electron charge, *n* the ideality factor, ϕ_B_ the zero-bias Schottky barrier height, and V is the applied voltage.

**Figure 3:**
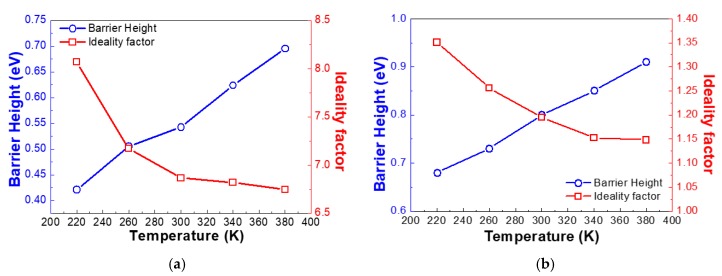
Plots of the barrier height and ideality factor as a function of temperature in (**a**) TE, and (**b**) the TFE model of the Si-based FS-GaN Schottky diode with Pd contact.

Figure 3a represents the extracted ideality factors and barrier heights of the Pd/Si-based FS-GaN Schottky diodes. It is essential to state that the inhomogeneity of the barrier height in the contact induces the gradient of the barrier height and the ideality factor of the diodes at ~260 K [19,20]. Furthermore, it was found that the drastic increment of current at 260 K in T-I-V curves also supports this behavior. This is commonly observable in Schottky diodes. Typically, most electrons cannot jump the barrier height in low temperatures since they do not have sufficient energy to leap it in a temperature-activated process, thus driving the lower barrier height values. However, the dominant electrons with a sufficient thermally activated energy can overcome the higher barrier height. This assigns the higher prevailing barrier height to a high temperature [21]. The TE model proposes that the ideality factors are distributed from 0.42 K to 0.70 K in the measured temperature area. Furthermore, the ideality factors in TE transport varied from 6.7 K to 8.2 K in a temperature range of 220–380 K. We attribute this to the spatially undulated barrier height in the contacts between the Pd contact and Si-based FS-GaN crystals, inherited from the surface defects embedded in the FS-GaN crystals [22,23]. In addition, these significant discrepancies of the ideality factors from the unity in the TE model definitely demonstrate that the charge transport mechanism of Si-based FS-GaN Schottky diodes includes another electronic tunneling conduction, such as thermionic field emission (TFE), field emission (FE), and multi-step tunneling. Given that TFE governs the tunneling conduction of the devices, the charge transport can be expressed as follows [24,25]:

(3) $$I=I_{0}exp\left(\frac{qV}{E_{00}coth\left(E_{00}/kT\right)}\right)=I_{0}exp\left(\frac{qV}{E_{0}}\right)$$

(4) $$I_{0}=\frac{AA^{**}T\sqrt{\pi E_{00}q\left(\phi_{B}-V-\xi \right)}}{kcosh\left(E_{00}/kT\right)}\times exp\left(-\frac{q\xi }{kT}-\frac{q\left(\phi_{B}-\xi \right)}{E_{00}coth\left(E_{00}/kT\right)}\right)$$

where *E*_00_ = (*q*ħ/2) (N_D_/m*ɛ_s_)^1/2^, *V*, *ξ*, *h*, m*, and *ε_s_* indicate the characteristic energy related to the tunneling probability of a potential barrier, the applied bias voltage, E_C_-E_F_, corresponding to *kT*/*q*ln(N_C_/N_D_), where N_C_ is the effective density of states in the conduction band (*N_C_* = 2.53 × 10^18^ cm^−3^ in GaN) [26], the Planck’s constant, the effective mass, and the dielectric constant, respectively. *E*_0_ = *nkT* = *E*_00_ coth (*E*_00_/*kT*). The calculated Schottky barrier heights and ideality factors were 0.68–0.91 eV and 1.14–1.35 eV, respectively. The TFE conduction model provides a much closer value to the unity, indicating that Poole–Frenkel emission is a more plausible explanation for the tunneling mechanism of Pd/Si-based FS-GaN Schottky devices. This behavior is similar to the results of other studies [27,28].

**Figure 4:**
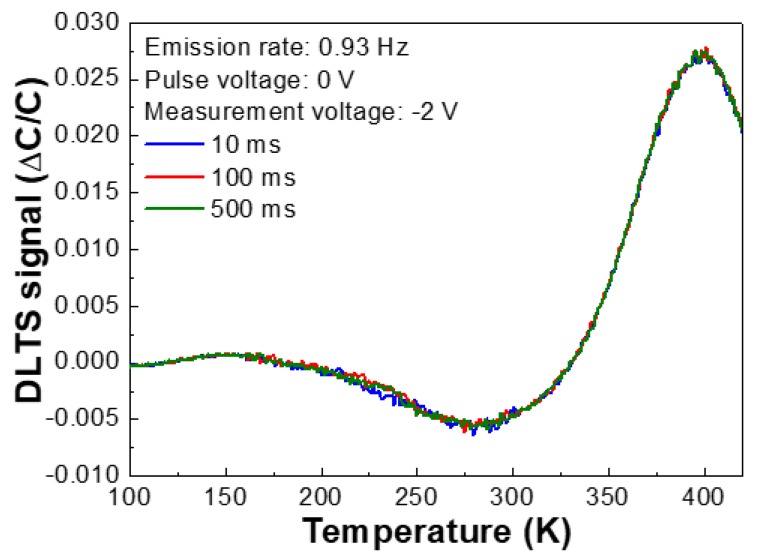
The DLTS spectra of Pd/Si-based GaN crystal Schottky diodes measured at the reverse voltage of −2 V in the temperature range of 100–420 K and in the pulsing time of 100–500 ms.

It is well established that tunneling, high series resistance, and interface states can give rise to the increased ideality factors or inhomogeneity of ϕ_B_. In addition, the growth characteristics of HVPE FS-GaN crystals exhibit a large quantity of nitrogen-related surface states, V_Ga_ point defect complex, and nitrogen anti-sites (N_Ga_) [29,30,31,32,33]. The charges can go through these tunneling sites and affect the characteristics of ideal Schottky diodes. Deep level transient spectroscopy (DLTS) measurements were employed to clarify the effects on conduction sites, as shown in Figure 4. Two deep trap levels were embedded into the FS-GaN stripped from a Si substrate, as shown in Figure 4. Table 1 summarizes the fingerprints of the electronic deep levels in the FS-GaN. The deep levels were positioned at ~0.24 eV, and ~1.06 eV below the conduction band edge. Non-variation of DLTS signals vs. pulsing time confirmed that these traps were related to the non-interacting point defects [34,35]. At low climate, the carriers of the deep levels built up, and released around 260 K, thereby augmenting the barrier heights. We must take into account that there was an abrupt increase of DLTS signals at 260 K. This indicates that the electronic deep trap carrier influenced the charge transport in Si-based FS-GaN. This agrees with the results of the T-I-V curves in Figure 2. Therefore, we believe that deep level defects embedded in Si-based FS-GaN can dominate the variation in Φ_B_ and n. One can obviously observe that the intensities of DLTS signals as a function of temperature are negligible, indicative of no variation of the trap concentrations [36]. It is noticeable that the characteristics of the electronic deep level traps in Si-based FS-GaN crystals are comparable to those of conventional FS-GaN (see Table 1). This clearly proves that the FS-GaN materials extracted from the in situ removal of substrate are desirable materials for futuristic applications.

## 4. Conclusions

The current transport mechanism of Pd/Si-based FS-GaN crystal Schottky diodes was investigated using the I-V-T analysis. The TE conduction model shows a larger deviation of the ideality factors from the unity, indicative of the involvement of another transport mechanism. We proved that the carrier transport in the Schottky diodes is dominated by the TFE conduction, not only the TE one. In addition, tunneling via non-interacting point defect complex plays a key role in charge transport, which was confirmed by the DLTS measurements. The electronic characteristics of the Pd/Schottky contacts to the Si-based FS-GaN layers stand comparatively with those of conventional FS-GaN grown using other methods. This study clearly suggests that the fabrication of Pd/Si-based FS-GaN Schottky diodes can provide a promising way to achieve GaN-based futuristic devices with high performances.

## Figures and Tables

**Table 1:** The defect parameters for the Pd/Si-based FS-GaN Schottky diodes and their comparison to other works.

Reference	Activation Energy (eV)	Capture Cross Section (cm^2^)	Trap Density (cm^−3^)
This study	0.24	1.65 × 10^−17^	1.07 × 10^14^
	1.06	1.76 × 10^−14^	2.19 × 10^15^
[37]	0.25, 0.53, 0.65, 0.69, 1.40, 1.55	10^−12^–10^−16^	~10^12^–2.2 × 10^15^
[38]	0.25, 0.35, 0.59, 0.66, 1.0	6.7 × 10^−14^–9.0 × 10^−16^	Mid-10^14^
[39]	0.6	2.0−10^−17^	-
